# Feature-indistinguishable machine unlearning via negative-hot label encoding and class weight masking

**DOI:** 10.1038/s41598-026-40379-9

**Published:** 2026-03-03

**Authors:** Jiali Wang, Hongxia Bie, Zhao Jing, Yichen Zhi

**Affiliations:** Intelligent Media Computing Center, School of Artificial Intelligence, Beijing University of Posts and Telecommunications, Beijing, 100876 China

**Keywords:** Machine unlearning, Label encoding, Feature indistinguishable representation, Computational biology and bioinformatics, Engineering, Mathematics and computing

## Abstract

With the growing importance of data privacy and regulatory compliance, machine unlearning has become a critical requirement in deep learning. However, existing approaches often require access to the original training data, incur substantial computational costs, or compromise performance on retained data. To address these limitations, we propose a novel unlearning framework that integrates label encoding fine-tuning with class weight masking, enabling efficient and selective forgetting of specific classes. In particular, we introduce Negative-Hot Label Encoding (NHLE), which suppresses the discriminability of target classes in the feature space, thereby weakening their representations. Our method requires only a small number of samples from the forgotten classes for iterative fine-tuning. Extensive experiments on multiple visual datasets show that the proposed framework achieves near-zero classification accuracy on forgotten data, while reducing accuracy on retained data by no more than 0.035.

## Introduction

Classification networks are fundamental to computer vision, supporting tasks such as image classification^1,2^, object detection^3,4^, semantic segmentation^5^, and face recognition^6^. These models have driven substantial progress in the field, yet they also raise critical concerns regarding data compliance and security. Regulatory frameworks such as the General Data Protection Regulation (GDPR)^7^ mandate the “right to be forgotten,” requiring models to accommodate situations where users revoke consent for data usage or where data collection is deemed inappropriate. Furthermore, retaining biased, obsolete, or adversarial data within models can undermine predictive performance and introduce significant security vulnerabilities. A central challenge, therefore, lies in eliminating the influence of specific data without resorting to complete retraining. In this context, machine unlearning has emerged as a promising paradigm. It seeks to selectively erase designated data through efficient parameter updates or targeted model adjustments, while safeguarding the integrity of the remaining knowledge and preserving model utility^8,9^.

In recent years, machine unlearning methods have generally been divided into two categories. The first comprises parameter update-based approaches, which achieve forgetting through weight correction, influence functions, or closed-form solutions^10–21^. The second includes loss function-based approaches, which reduce the influence of forgotten data by introducing distributional constraints, information-theoretic measures, or optimization in gradient and feature spaces^22–36^. Despite their different emphases, these approaches share several limitations. Many methods depend on access to the original training data; parameter update-based strategies often involve computationally expensive Hessian matrix calculations; and, in most cases, unlearning inevitably degrades the performance of retained data. Balancing the completeness of forgetting with computational efficiency and model utility therefore remains a central challenge in this field.

To reduce reliance on original data, recent studies have increasingly explored synthetic data generation and relabeling strategies as alternatives to direct access to the training set. One line of research produces substitute samples that approximate the distributional characteristics of the data to be forgotten, including error-maximization noise^37,38^, adversarial perturbations^10,11,17,32^, and counterfactual examples^29,39^. These substitutes are introduced during fine-tuning to facilitate effective erasure. Another line of work focuses on relabeling, where the data to be forgotten is assigned incorrect labels^39–41^ or pseudo-labels^42,43^. This encourages the model to deliberately “confuse” or “misremember” the targeted samples, thereby weakening their influence on the decision boundary. While these methods reduce dependence on original data and open new avenues for efficient and controllable unlearning, notable limitations persist. Generating synthetic data or perturbations may introduce additional computational overhead, relabeling cannot fully remove the effects of forgotten data, and a trade-off remains between the completeness of forgetting and the preservation of performance on retained data.

To address these challenges, this study proposes an innovative machine unlearning approach. We introduce a novel unlearning label encoding scheme and employ a small number of samples from the forgotten class for fine-tuning, ensuring that the target class becomes indistinguishable from others in the feature space. By further integrating class-wise weight masking^44^, the method achieves thorough forgetting of designated data while maximally preserving the performance of retained data. Compared with traditional methods, the proposed approach removes the need for access to the complete original dataset and avoids computationally expensive high-order matrix operations, while maintaining a favorable balance between forgetting and preservation. In summary, it offers a new technical pathway for efficient and reliable machine unlearning.

The main contributions of this work are as follows:

1. We propose a novel machine unlearning method that combines label encoding-based fine-tuning with class-wise weight masking, enabling efficient and controllable forgetting in deep models.
2. We introduce the Negative-Hot Label Encoding (NHLE) strategy, which enforces indistinguishability between the target forgotten classes and other classes in the feature space, effectively weakening their representational capacity.
3. Extensive experiments on multiple datasets demonstrate that the proposed method achieves superior forgetting effectiveness while maintaining the performance of retained data.

## Related work

In the study of machine unlearning, existing methods can be broadly classified into three categories based on their technical implementation.

**Parameter update-based methods.** These approaches achieve unlearning by directly modifying model parameters. Techniques include selectively suppressing parameters associated with the forgotten data^10–12,20^, approximating the reversal of the training process via influence functions or closed-form solutions^13,15–18^, and accelerating model updates through training caches or fine-tuning strategies^14,19,21^. These methods efficiently reduce the impact of forgotten data. However, they generally still require access to the original training set and often involve computationally intensive operations, such as Hessian matrix calculations.

**Loss function-based methods.** These approaches achieve unlearning by designing objective functions that limit the influence of forgotten data. Techniques include distribution alignment or information-theoretic metrics^22–24,27,31,34^, Bayesian or regularization-based constraints^25,28,36^, and optimization of gradients or centroid distances^26,30,33,35^. These methods focus on selective forgetting while maintaining performance on retained data. However, they still heavily depend on access to the original training set and often require extensive retraining.

**Synthetic data- or relabeling-based methods.** To reduce reliance on the original training data, some studies introduce generated substitute samples, such as error-maximization noise, adversarial perturbations, or counterfactual examples^10,17,27,29,32,37–39^, or relabel the forgotten data with incorrect or pseudo labels^39–43^, actively confusing these samples during training. These approaches provide greater control over the forgetting process while maintaining effectiveness; however, a trade-off remains between the completeness of forgetting and the performance of retained data.

These approaches improve forgetting effectiveness and reduce reliance on the original data; however, a trade-off between the completeness of forgetting and the performance on retained data remains. In contrast, class-wise weight masking^44^ does not depend on global parameter information, offering higher computational efficiency while effectively preserving model performance on retained data. Nevertheless, because the features of forgotten data remain distinguishable from those of retained data, its capacity for thorough forgetting is limited. To overcome this limitation, the method proposed in this study combines fine-tuning with a small number of forgotten samples and class-wise weight masking, achieving efficient and controllable complete forgetting while maximizing the performance of retained data.

## Achieving feature indistinguishable through label encoding and weight masking

### Framework overview

This section presents our proposed unlearning method for machine learning. The core idea is to reduce the feature separability of forgotten categories through stepwise fine-tuning based on label encoding, while simultaneously suppressing their influence on class decisions via per-class weight masking. This two-step approach effectively removes forgotten categories at both the feature and decision levels, while preserving the model’s ability to learn from retained data.

Specifically, we construct a unified framework consisting of two complementary steps:

1. Negative-Hot Label Encoding (NHLE): This step weakens the representational strength of the forgotten categories in the feature space, making them indistinguishable from other categories.
2. Class Weight Masking (CWM): This step suppresses the influence of the forgotten categories on the final class predictions, ensuring that their residual impact on decision-making is minimized.

**Fig. 1:**
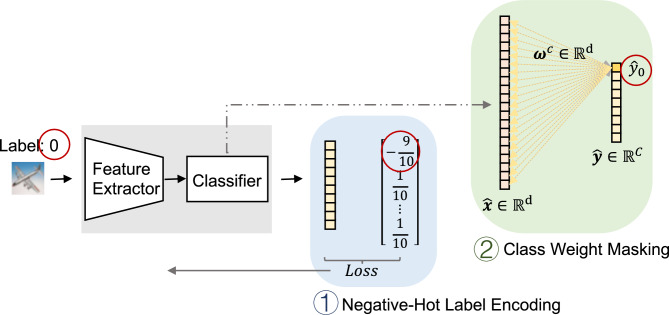
Overview of the proposed NHLE–CWM machine unlearning framework. Step 1: Negative-Hot Label Encoding (NHLE), where the forgotten class 0 is assigned a negative value, $-9/10$, and all other classes are assigned a positive value, 1/10. Step 2: Class Weight Masking (CWM).

These two steps work synergistically to form the complete NHLE–CWM framework, the structure of which is illustrated in Fig. 1.

First, Negative-Hot Label Encoding (NHLE) is introduced to restructure the label space. Traditional one-hot encoding reinforces the discriminability of the target class; however, in unlearning scenarios, this property hinders complete forgetting. To address this issue, NHLE incorporates a negative-hot mechanism that assigns negative encodings to the forgotten classes, thereby weakening or even suppressing their feature discriminability. This strategy progressively blurs the feature representations of forgotten classes and increases their indistinguishability from the retained data, ultimately enhancing the overall effectiveness of unlearning.

Second, Class Weight Masking (CWM) operates at the decision level by selectively masking weights in the model that are strongly associated with the forgotten classes, preventing them from influencing the inference process. Compared with approaches that rely solely on label encoding, CWM more directly severs the impact of forgotten classes on the model’s outputs, effectively eliminating their discriminative influence during decision-making. This mechanism not only reinforces the completeness of forgetting but also reduces interference with the learning and performance of retained classes.

By combining NHLE and CWM, the proposed method achieves synergistic effects at both the feature and decision levels. NHLE focuses on weakening the separability of forgotten classes during the input and feature representation stages, while CWM further eliminates their residual influence during the discrimination and output stages. These two components are complementary, enabling the NHLE–CWM framework not only to enhance the effectiveness and completeness of forgetting but also to maintain a balance between forgetting and retention, thereby ensuring controllability of forgetting while preserving overall model performance. In the following sections, we provide a detailed exposition of the specific design of NHLE, its theoretical basis, and the overall optimization strategy of the proposed method.

### Negative-hot label encoding (NHLE)

To achieve category-specific forgetting, we examine the model training process at the feature representation level, focusing on how the model parameters encode and retain the characteristics of the samples to be forgotten.

In a typical classification neural network, the architecture generally comprises a feature extractor and a classifier. The classifier usually consists of a fully connected layer followed by a Softmax activation function. During training, data labels are commonly represented using one-hot encoding, which guides the feature extractor to learn discriminative feature representations and enables the classifier to perform accurate class discrimination.

When a sample from class 0 is input, the model extracts a feature vector denoted as $\textbf{h}_0$, which is then passed to the classifier to generate a prediction. Specifically, given the classifier weights $\boldsymbol{\omega }$ and bias $\textbf{b}$, the output is computed as:

1 $$\begin{aligned} \hat{\textbf{y}} = \text {softmax}(\boldsymbol{\omega } \textbf{h}_0 + \textbf{b}) \end{aligned}$$

During backpropagation, the gradient of the classifier weights is given by:

2 $$\begin{aligned} \nabla \boldsymbol{\omega } = (\hat{\textbf{y}} - \textbf{y}) \textbf{h}_0^\top \end{aligned}$$

where $\textbf{y}$ denotes the one-hot encoded label vector. At the initial stage of training, when the model has not yet learned to differentiate between categories, its predictions can be approximated by a uniform distribution: $\hat{\textbf{y}} = \left[ \frac{1}{C}, \frac{1}{C}, \dots , \frac{1}{C}\right] ^\top$, where *C* is the total number of categories. Under this assumption, for a sample from class 0, the gradient can be expressed as:

3 $$\begin{aligned} \nabla \boldsymbol{\omega } = \begin{bmatrix} \frac{1}{C} - 1 \\ \frac{1}{C} \\ \vdots \\ \frac{1}{C} \end{bmatrix} h_0^\top \end{aligned}$$

**Fig. 2:**
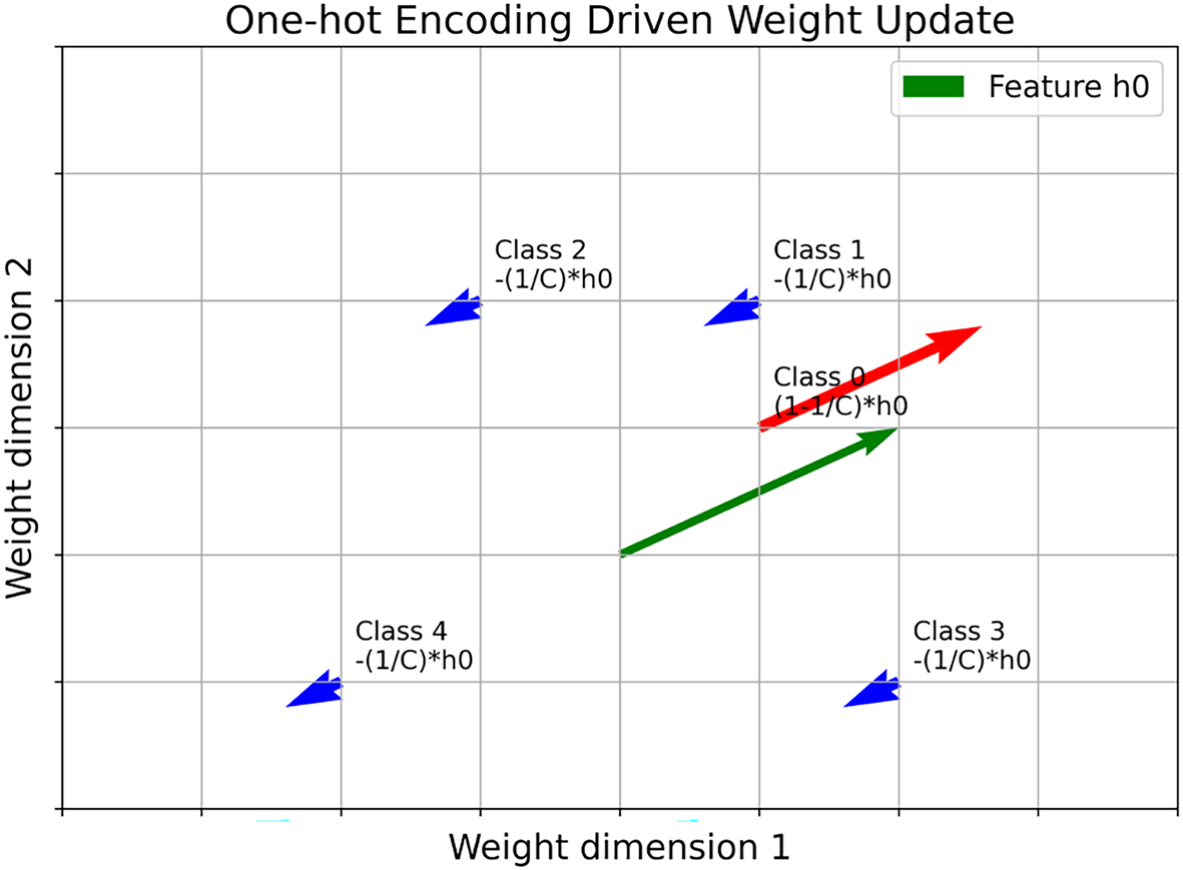
Illustration of gradient updates under one-hot encoding. The target class (class 0) receives a strong positive update, reinforcing its alignment with the corresponding class weight, while the non-target classes receive smaller negative updates, enhancing the separability of the target class in the parameter space.

This indicates that one-hot encoding strengthens the alignment of the feature vector $\textbf{h}_0$ with its corresponding class weight by a magnitude of $1 - \frac{1}{C}$, while applying a weaker opposite adjustment of $\frac{1}{C}$ to the other class weights, as shown in Fig. 2. Consequently, one-hot encoding inherently enhances the separability of the target class in parameter space.

As analyzed above, the one-hot encoding scheme continuously reinforces the discriminability of the target class in the parameter space during training, making it unsuitable for unlearning tasks. In contrast, our objective is to weaken the contribution of the forgotten class and gradually eliminate its representation. Motivated by this, we propose Negative-Hot Label Encoding (NHLE). Unlike one-hot encoding, which assigns a positive activation to the target class, NHLE assigns a negative weight to the forgotten class while uniformly distributing positive weights across the remaining classes. This design produces gradient updates opposite to those of one-hot encoding, actively suppressing the feature representations of the forgotten class while preserving the discriminability of the other classes.

Formally, in a *C*-class classification task, let $c \in \{0, 1, \ldots , C-1\}$ denote the class to be forgotten. The NHLE label vector $\tilde{\textbf{y}}^{(c)} \in \mathbb {R}^C$ is defined as:

4 $$\begin{aligned} \tilde{y}_j^{(c)} = {\left\{ \begin{array}{ll} \frac{1}{C} - 1, & j = c \\ \frac{1}{C}, & j \ne c \end{array}\right. } \end{aligned}$$

This definition explicitly suppresses the forgotten class during training, while assigning balanced positive weights to the remaining classes, forming a distinctive encoding scheme that combines “negative-hot” with “positive-cold” in the label space. Assigning a negative weight to the forgotten class actively weakens its influence on parameter updates during backpropagation, thereby facilitating effective unlearning. Simultaneously, the uniform positive weights across the other classes prevent excessive bias toward any single class, ensuring that decision boundaries remain stable and the discriminability of non-forgotten classes is preserved. The step-wise NHLE procedure is summarized in Algorithm 1.

**Algorithm 1:**
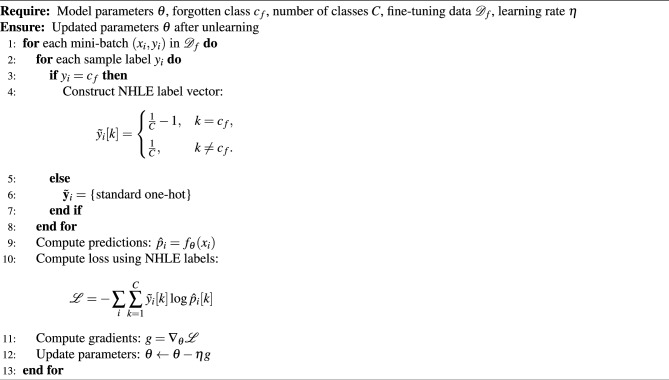
Negative-Hot Label Encoding (NHLE) for Class-Specific Unlearning

Assuming that the classification network is fully trained, the predicted vector for a class-0 sample can be approximated as: $\hat{\textbf{y}}^{(0)} \approx \big [1-\epsilon , \frac{\epsilon }{C-1}, \frac{\epsilon }{C-1}, \dots , \frac{\epsilon }{C-1}\big ]$, where $\epsilon$ is a small positive number close to zero, representing the minor probability mass assigned to non-target classes. When forgetting class-0 data using the NHLE encoding, the resulting gradient is:

5 $$\begin{aligned} \nabla \boldsymbol{\omega } = \begin{bmatrix} 2-\frac{1}{C} - \epsilon \\ \frac{\epsilon }{C-1}-\frac{1}{C} \\ \vdots \\ \frac{\epsilon }{C-1}-\frac{1}{C} \end{bmatrix} h_0^\top \end{aligned}$$

NHLE generates an update signal that not only reverses the direction of the original gradient but also amplifies its magnitude for the forgotten class, effectively “pushing back” the previously reinforced representation. In contrast, the gradient components of non-target classes remain relatively small, approximately $\frac{1}{C}$, indicating that NHLE exerts only mild suppression on these classes while preserving inter-class equilibrium. Compared with Equation (3), NHLE induces a gradient update direction opposite to that of standard one-hot encoding in the parameter space, thereby eliminating the residual influence of the forgotten data from the network.

For comparison, if a simpler label is used for the class-0 sample, $\textbf{y}_0^{(B)} = [-1, 0, 0, \dots , 0]$, the gradient becomes:

6 $$\begin{aligned} \nabla \boldsymbol{\omega } = \begin{bmatrix} 2-\epsilon \\ \frac{\epsilon }{C-1} \\ \vdots \\ \frac{\epsilon }{C-1} \end{bmatrix} h_0^\top \end{aligned}$$

**Fig. 3:**
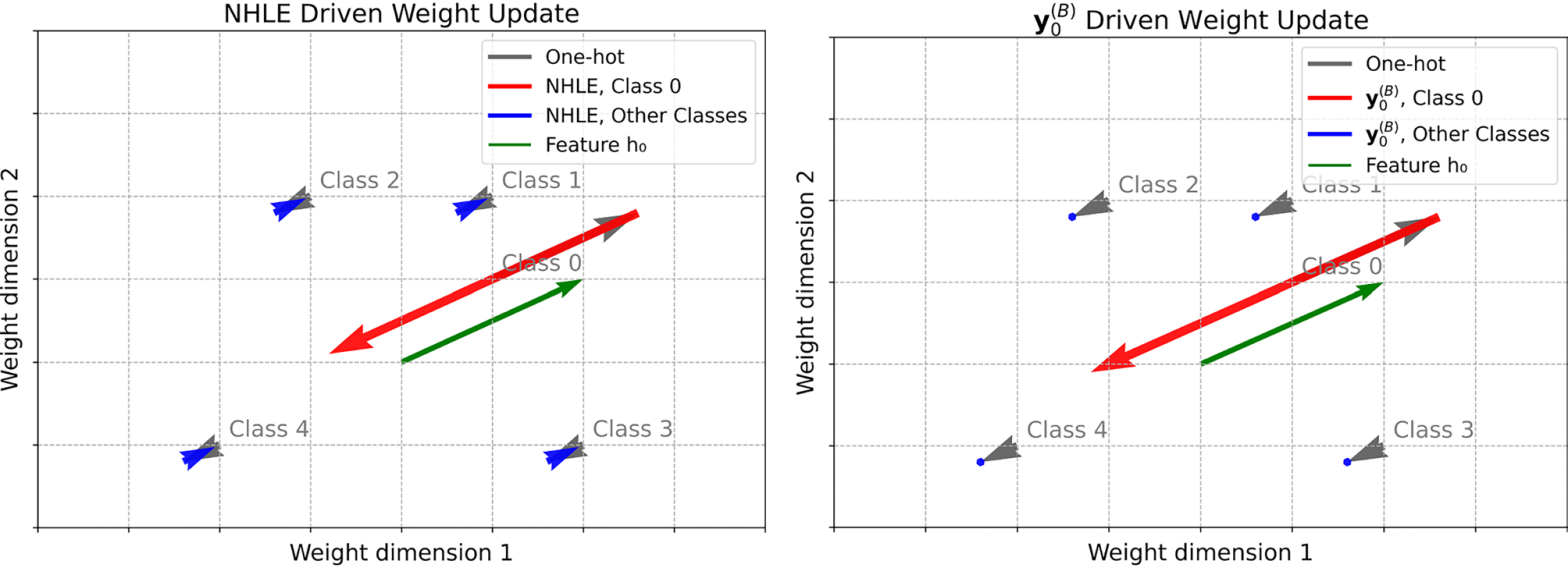
Comparison of gradient update directions for NHLE and $\textbf{y}_0^{(B)}$. The key difference is that NHLE provides mild negative gradients for non-target classes, while $\textbf{y}_0^{(B)}$ yields nearly zero gradients (blue arrow).

The gradient components for class 0 approaches 2, effectively suppressing the previously reinforced representation of the forgotten class. However, the gradient components for non-target classes remain approximately zero, meaning that this encoding cannot eliminate residual influence on other class parameters and may potentially degrade the performance of retained data. The contrasting update directions for NHLE and this simple label encoding are illustrated in Fig. 3.

The name NHLE is directly inspired by the concept of one-hot encoding. In one-hot encoding, the target class is assigned a value of 1 (“hot”), while all other classes are set to 0 (“cold”). In contrast, NHLE assigns a negative value to the position corresponding to the class to be forgotten–hence “negative-hot”–to explicitly suppress its contribution during training. Simultaneously, positive values are uniformly distributed across the remaining classes to preserve the discriminability of the classification boundaries. Here, the term “hot” still denotes the emphasized class dimension in the vector, while the prefix “negative” highlights its suppressive role in the learning process.

**Fig. 4:**
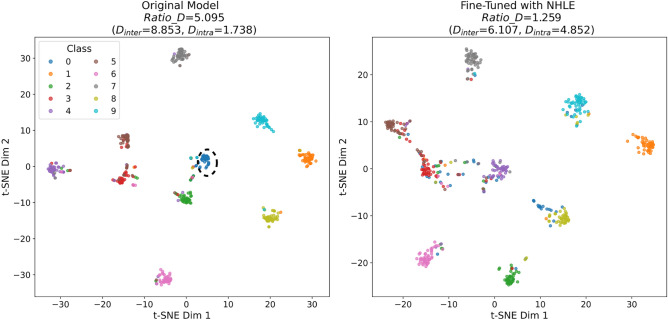
Visualization of feature representations for the CIFAR10 dataset when forgetting class 0 using VGG16. Left: Under traditional one-hot encoding, class 0 forms a distinct cluster (indicated by the black dashed circle). Right: Under NHLE, its features gradually overlap with those of other classes.

To further illustrate the effect of the proposed NHLE on learned representations, we conducted a feature visualization experiment in a simplified setting (see Fig. 4). Specifically, we extracted the activations from the penultimate layer of the network and applied t-SNE to project them into a two-dimensional space. Under conventional one-hot encoding, the forgotten class still forms a distinct and compact cluster in the feature space. In contrast, when NHLE is applied, its features gradually merge with those of other classes, becoming indistinguishable. Meanwhile, the feature clusters of the non-forgotten classes remain well separated.

These observations are consistent with our theoretical analysis and intuitively demonstrate that NHLE effectively suppresses the discriminability of the forgotten class while preserving the separability of the retained ones.

### NHLE-CWM for feature-indistinguishable unlearning

The proposed NHLE actively obfuscates the feature representations of forgotten samples during fine-tuning, reducing their separability in the feature space. However, this approach faces a common limitation inherent to label-layer modifications: because the parameters of the early and intermediate layers are influenced by data from multiple classes, fine-tuning inevitably affects the performance on retained data. In practice, the number of forgotten samples and the selection of hyperparameters must be carefully balanced. Using a large number of samples can achieve effective forgetting while preserving retained data performance, but the training cost approaches that of retraining the entire model. Conversely, relying on a small number of samples may degrade the performance on retained data. Thus, striking a balance between effective forgetting and maintaining retained performance remains a challenging problem.

Class Weight Masking (CWM) is a machine unlearning technique designed to suppress a model’s ability to discriminate forgotten data by masking weights associated with specific classes. Traditional methods based on parameter updates or influence functions often rely on global parameter information or Hessian matrix computations, resulting in high computational costs and potential degradation of performance on retained data. In contrast, CWM selectively masks only the weights of the targeted classes, minimizing impact on retained data and preserving model performance. This targeted approach achieves higher computational efficiency while maintaining effectiveness in unlearning.

Building on this motivation, we propose the NHLE-CWM method, which integrates Negative-Weighted Label Encoding (NHLE) with Class Weight Masking (CWM) to achieve efficient and controllable complete unlearning. The method comprises two steps. First, NHLE is applied to the forgotten data in the label space by assigning the forgotten class a weight of $\frac{1}{C} - 1$ and the remaining classes a weight of $\frac{1}{C}$, guiding the model to reduce the separability between forgotten and retained data during feature extraction. Second, CWM is employed to mask the corresponding class weights, further suppressing the model’s ability to discriminate the forgotten class. Through fine-tuning on a small number of forgotten samples, this synergistic mechanism effectively diminishes the influence of forgotten data in both the feature and decision spaces while maximizing the preservation of performance on retained data.

The synergy between NHLE and CWM is central to the efficiency of our unlearning method: NHLE reduces the discriminability of forgotten data at the feature level, while CWM further mitigates its impact at the decision level. This combination allows the model to selectively unlearn while preserving the integrity of the original knowledge. Moreover, the method requires only a small number of forgotten samples for fine-tuning and does not need access to the full original training set, providing significant advantages in data compliance and computational efficiency. In summary, the NHLE-CWM approach enhances the completeness of unlearning while maintaining performance on retained data, offering a scalable and practical solution for efficient and controllable machine unlearning.

## Experiment

### Experiment setting

The experiments were conducted on five benchmark datasets: MNIST, FashionMNIST, SVHN, CIFAR10, and CIFAR100. To evaluate the applicability of the proposed method across different model architectures, various neural networks were employed, including MLP, LeNet, VGG, ResNet, InceptionV3 and MobileNetV3-L. For the unlearning tasks, both single-class and multi-class forgetting experiments were designed. In the 10-class tasks, single-class forgetting involved removing class 0, while multi-class forgetting involved removing classes 0 and 4. In the 100-class tasks, multi-class forgetting was implemented by randomly removing 20 classes. This experimental setup simulates forgetting requirements of varying scales and complexities, providing a comprehensive evaluation of the method’s performance.

In the single-class forgetting experiments, only 16 samples from the forgotten class were used to fine-tune the model. For the multi-class forgetting experiments, 8 samples from each class to be forgotten were selected for fine-tuning. The samples were not specially chosen but were taken sequentially from the datasets. This design ensures a small and easily manageable fine-tuning set while effectively assessing the unlearning method’s performance under limited sample conditions.

In the forgetting task, model performance is assessed using the classification accuracies of the forgotten and retained classes, denoted as Acc_F and Acc_R, respectively. Effective forgetting is reflected by a low Acc_F alongside a high Acc_R. In the feature non-separability experiment, the separability of feature representations is quantified by measuring the inter-class distance, intra-class distance, and their ratio. We denote the distance between the forgotten class and all retained classes as $D_{inter}$, where a smaller value indicates that the forgotten class has moved closer to the other classes in the feature space. The intra-class distance of the forgotten class is denoted as $D_{intra}$, where a larger value signifies a more dispersed distribution of its feature representations. Accordingly, a smaller ratio $Ratio\_D=\frac{D_{inter}}{D_{intra}}$ implies that the forgotten class exhibits reduced discriminative power relative to the retained classes, reflecting stronger non-separability in the feature space.

### Effectiveness of single-class unlearning

We evaluated NHLE-CWM on single-class unlearning using 16 samples from the forgotten class (class 0). As shown in Table 1, NHLE-CWM effectively reduces Acc_F to nearly zero across different datasets and models, while exerting only minimal impact on Acc_R. On MNIST with an MLP, Acc_F drops to 0.000 and Acc_R decreases slightly from 0.985 to 0.957 ($\Delta$)Acc_R = -0.028), representing the largest performance loss observed and highlighting the method’s robustness. On FashionMNIST with LeNet, Acc_F reaches 0.000 and Acc_R even increases by 0.001, demonstrating thorough forgetting with negligible or positive effects on retained class performance. For more complex datasets such as CIFAR-10, small but nonzero Acc_F values arise due to richer feature representations and greater intra-class variability, which make complete forgetting more challenging compared to simpler datasets like MNIST.

Compared with state-of-the-art methods on CIFAR10 and CIFAR100 (Table 2), NHLE-CWM consistently achieves lower Acc_F while better preserving Acc_R. For example, on CIFAR10 with VGG16, NHLE-CWM reduces Acc_F to 0.025 with only a slight drop in Acc_R ( $\Delta$ Acc_R = -0.009), while GKT and WF-Net incur greater drops in Acc_R. Similar trends are observed on CIFAR100.

These results demonstrate that NHLE-CWM enables effective single-class forgetting, achieving a favorable trade-off between erasing the forgotten class and preserving retained data, even when using a very small fine-tuning set.

**Table 1:** Single-class unlearning results of NHLE-CWM across different datasets and models. Acc_R and Acc_F denote the classification accuracy of retained and forgotten classes, respectively, and $\Delta$Acc_R represents the change in retained class accuracy after unlearning.

Dataset	Model	Original network		Unlearning network		$\Delta$Acc_R$\uparrow$
		Acc_R	Acc_F	Acc_R	Acc_F$\downarrow$	
MNIST	MLP	0.985	0.994	0.957	0.000	-0.028
	LeNet	0.991	0.996	0.976	0.000	-0.015
FashionMNIST	MLP	0.905	0.862	0.890	0.000	-0.015
	LeNet	0.908	0.854	0.909	0.000	0.001
SVHN	VGG11	0.957	0.966	0.957	0.000	0.000
	ResNet18	0.962	0.966	0.956	0.001	-0.006
CIFAR10	VGG16	0.895	0.928	0.886	0.025	-0.009
	ResNet34	0.891	0.910	0.891	0.067	0.000
	InceptionV3	0.933	0.938	0.928	0.014	-0.005
	MobileNetV3L	0.805	0.846	0.787	0.000	-0.018
CIFAR100	VGG16	0.650	0.860	0.627	0.000	-0.023
	ResNet50	0.661	0.880	0.639	0.000	-0.022
	InceptionV3	0.752	0.880	0.734	0.000	-0.018

**Table 2:** Comparison of single-class unlearning performance on CIFAR10 and CIFAR100 between NHLE-CWM and existing methods.

Dataset	Method	Model	Original network		Unlearning network		$\Delta$Acc_R$\uparrow$
			Acc_R	Acc_F	Acc_R	Acc_F$\downarrow$	
CIFAR10	PBU^25^	ResNet18	0.765	0.718	0.662	0.045	-0.103
		AllCNN	0.849	0.796	0.762	0.010	-0.087
		ResNet34	0.768	0.685	0.686	0.006	-0.081
	GKT^37^(zero-shot)	AllCNN	0.941	0.875	0.820	0.000	-0.121
		LeNet	0.598	0.623	0.413	0.000	-0.185
		ResNet9	0.848	0.885	0.568	0.000	0.280
	NG-IR^38^	ResNet18	0.779	0.810	0.716	0.000	-0.063
		AllCNN	0.826	0.910	0.739	0.000	-0.087
	WF-Net^35^	VGG16	0.930	0.930	0.802	0.183	-0.128
		ResNet18	0.939	0.940	0.797	0.093	-0.142
		ViT-T	0.780	0.780	0.735	0.000	-0.045
	**NHLE-CWM**(Ours)	VGG16	0.895	0.928	0.886	**0.025**	**-0.009**
		ResNet34	0.891	0.910	0.891	**0.067**	**0.000**
		InceptionV3	0.933	0.938	0.928	**0.014**	**-0.005**
		MobileNetV3L	0.805	0.846	0.787	**0.000**	**-0.018**
CIFAR100	PBU^25^	Resnet18	0.761	0.801	0.696	0.015	-0.065
		Resnet50	0.760	0.784	0.693	0.003	-0.067
		Resnet34	0.752	0.840	0.653	0.002	-0.099
	NG-IR^38^	ResNet18	0.787	0.830	0.754	0.000	-0.033
		MobileNetV2	0.774	0.900	0.758	0.000	-0.016
	WF-Net^35^	VGG16	0.930	0.930	0.802	0.183	-0.128
		ResNet18	0.939	0.940	0.797	0.093	-0.142
		ViT-T	0.780	0.780	0.735	0.000	-0.045
	**NHLE-CWM**(Ours)	VGG16	0.650	0.860	0.627	**0.000**	**-0.023**
		ResNet50	0.661	0.880	0.639	**0.000**	**-0.021**
		InceptionV3	0.752	0.880	0.734	**0.000**	**-0.018**

### Effectiveness of multi-class forgetting

We further evaluated NHLE-CWM on multi-class unlearning, where 8 samples from each forgotten class were used for fine-tuning. The results in Table 3 are reported as the mean ± standard deviation over 30 independent runs, confirming the robustness of NHLE-CWM. As shown, NHLE-CWM consistently reduces Acc_F to nearly zero while maintaining a high level of Acc_R. For example, on CIFAR10 with ResNet34, Acc_R decreases slightly from 0.892 to 0.877 ($\Delta$Acc_R = -0.014). On CIFAR100 with VGG16, Acc_R increases from 0.664 to 0.672 ($\Delta$Acc_R = 0.008), indicating that the forgetting process is not only thorough but may also bring marginal benefits to retained classes. Furthermore, Table 4 compares NHLE-CWM with NG-IR on CIFAR10 and CIFAR100, showing that NHLE-CWM consistently achieves lower Acc_F and better preserves Acc_R across all evaluated models.

These results demonstrate that NHLE-CWM remains effective in multi-class forgetting scenarios and achieves efficient, controllable unlearning even with a small fine-tuning set. The method balances thorough forgetting of multiple classes with the preservation of retained data performance, validating its applicability to more complex unlearning tasks.

**Table 3:** Multi-class unlearning results of NHLE-CWM across different datasets and models.

Dataset	Model	Original network		Unlearning network		$\Delta$Acc_R$\uparrow$
		Acc_R	Acc_F	Acc_R	Acc_F$\downarrow$	
MNIST	MLP	0.985	0.988	0.950+0.020	0.000+0.000	-0.035
	LeNet	0.990	0.995	0.971+0.004	0.000+0.000	-0.019
FashionMNIST	MLP	0.912	0.854	0.888+0.032	0.000+0.000	-0.024
	LeNet	0.917	0.846	0.893+0.015	0.000+0.000	-0.024
SVHN	VGG11	0.956	0.964	0.931+0.022	0.000+0.000	-0.025
	ResNet18	0.961	0.969	0.960+0.002	0.000+0.000	-0.001
CIFAR10	VGG16	0.895	0.914	0.882+0.062	0.000+0.000	-0.013
	ResNet34	0.892	0.898	0.877+0.092	0.000+0.000	-0.014
	InceptionV3	0.931	0.943	0.903+0.038	0.002+0.000	-0.028
	MobileNetV3L	0.803	0.835	0.796+0.021	0.000+0.000	-0.007
CIFAR100	VGG16	0.664	0.604	0.672+0.001	0.000+0.000	0.008
	ResNet50	0.676	0.609	0.680+0.001	0.000+0.000	0.004
	InceptionV3	0.760	0.725	0.733+0.004	0.000+0.000	-0.027

**Table 4:** Comparison of multi-class unlearning performance on CIFAR10 and CIFAR100 between NHLE-CWM and existing methods.

Dataset	Method	Model	Original network		Unlearning network		$\Delta$Acc_R$\uparrow$
			Acc_R	Acc_F	Acc_R	Acc_F$\downarrow$	
CIFAR10	NG-IR^38^	ResNet18	0.780	0.787	0.736	0.000	-0.044
		AllCNN	0.843	0.797	0.808	0.000	-0.035
	**NHLE-CWM**(Ours)	VGG16	0.895	0.914	0.886	**0.000**	**-0.009**
		ResNet34	0.892	0.898	0.885	**0.000**	**-0.007**
		InceptionV3	0.931	0.943	0.920	**0.000**	**-0.011**
		MobileNetV3L	0.803	0.835	0.805	**0.000**	**0.002**
CIFAR100	NG-IR^38^	ResNet18	0.779	0.828	0.754	0.000	-0.025
		MobileNetV2	0.765	0.817	0.763	0.000	-0.002
	**NHLE-CWM**(Ours)	VGG16	0.664	0.604	0.675	**0.000**	**0.011**
		ResNet50	0.676	0.609	0.683	**0.000**	**0.007**

### Comparative analysis of label encodings

To further validate the effectiveness of NHLE, we conducted a comparative experiment using a simpler label encoding scheme, $\textbf{y}_0^{(B)} = [-1, 0, 0, \dots , 0]$. As shown in Table 5, NHLE successfully forgets the target class while maintaining strong classification performance on the retained classes. In contrast, the simpler encoding scheme leads to a notable degradation in the performance of the retained data. These results reinforce the theoretical justification provided in the “Negative-Hot Label Encoding (NHLE)” section and demonstrate the practical advantages of the proposed method.

**Table 5:** Performance comparison of NHLE and simple label encoding.

Method	Accuracy		Representation separability		
	Acc_R $\uparrow$	Acc_F $\downarrow$	$D_{inter} \downarrow$	$D_{intra} \uparrow$	$Ratio\_D \downarrow$
Original Network	0.895	0.928	8.853	1.738	5.095
NHLE	**0.886**	0.496	6.551	4.557	1.438
$\textbf{y}_0^{(B)}$	**0.348**	0.000	3.024	3.912	0.773

### Ablation study on component synergy

To illustrate the individual and joint effects of NHLE and CWM, we conducted ablation experiments on CIFAR10 using VGG16. Table 6 reports results under three settings: NHLE only, CWM only, and their combination. NHLE alone reduces $D_{inter}$ and increases $D_{intra}$, making the features of the forgotten class nearly indistinguishable. CWM alone achieves a more substantial reduction in Acc_F. When combined, NHLE and CWM not only maintain the forgetting performance but also make the features of both forgotten and retained classes indistinguishable, demonstrating their complementary and synergistic effects.

**Table 6:** Ablation study of NHLE and CWM on CIFAR10 (VGG16).

Configuration	Accuracy		Representation separability		
	Acc_R $\uparrow$	Acc_F $\downarrow$	$D_{inter} \downarrow$	$D_{intra} \uparrow$	$Ratio\_D \downarrow$
Original Network	0.895	0.928	8.853	1.738	5.095
NHLE	0.886	0.496	**6.551**	**4.557**	**1.438**
CWM	0.895	**0.025**	8.853	1.738	5.095
NHLE+CWM	0.886	**0.025**	**6.551**	**4.557**	**1.438**

### Feature representation inseparability analysis

This experiment evaluates the impact of NHLE-based fine-tuning on the separability of feature representations in the CIFAR-10 dataset. For models fine-tuned with different learning rates, we computed $D_{inter}$, $D_{intra}$, and their ratio $Ratio\_D$, and additionally assessed classification performance using Acc_F and Acc_R. The learning rate ranged from 0.10 to 0.20 with a step size of 0.01.

**Fig. 5:**
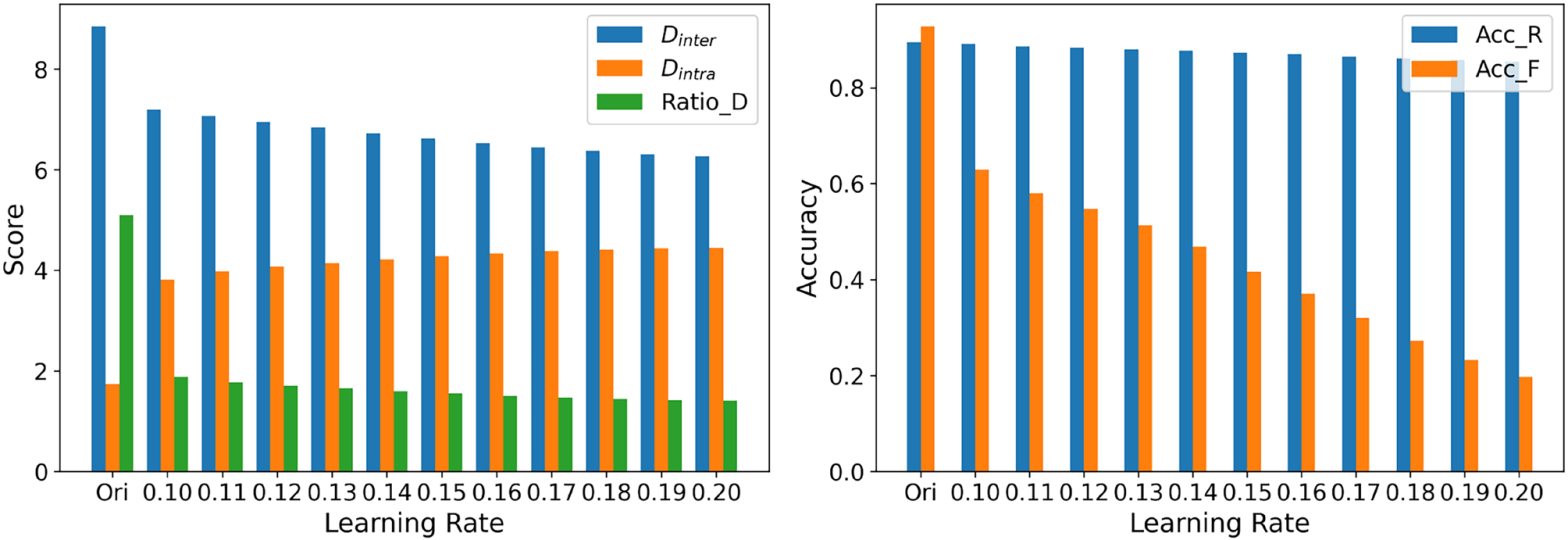
Left: Inter-class and intra-class score and their ratio for the forgotten class at different learning rates. Right: Classification accuracy of the forgotten class and retained classes.

As shown in Fig. 5 (left: feature separability metrics; right: classification performance), the first group of bars corresponds to the original model before fine-tuning. After fine-tuning, all models exhibit a consistent pattern: $D_{inter}$ decreases substantially, $D_{intra}$ increases, and consequently $Ratio\_D$ drops markedly. This indicates that features of the forgotten class become less distinguishable from those of the retained classes, accompanied by a reduction in Acc_F.

It is worth noting that, although models with different learning rates still show some variation, the magnitude of these differences is much smaller than the overall change induced by fine-tuning itself. As the learning rate increases, both $Ratio\_D$ and Acc_F exhibit a gradual downward trend, suggesting that larger learning rates further suppress feature separability, but only in a moderate and incremental manner. Overall, the influence of the learning rate mainly affects the fine-tuning degree, whereas the core NHLE effect–namely, reducing the separability of the forgotten class–remains stable across learning rates.

### t-SNE visualization of forgotten class predictions

**Fig. 6:**
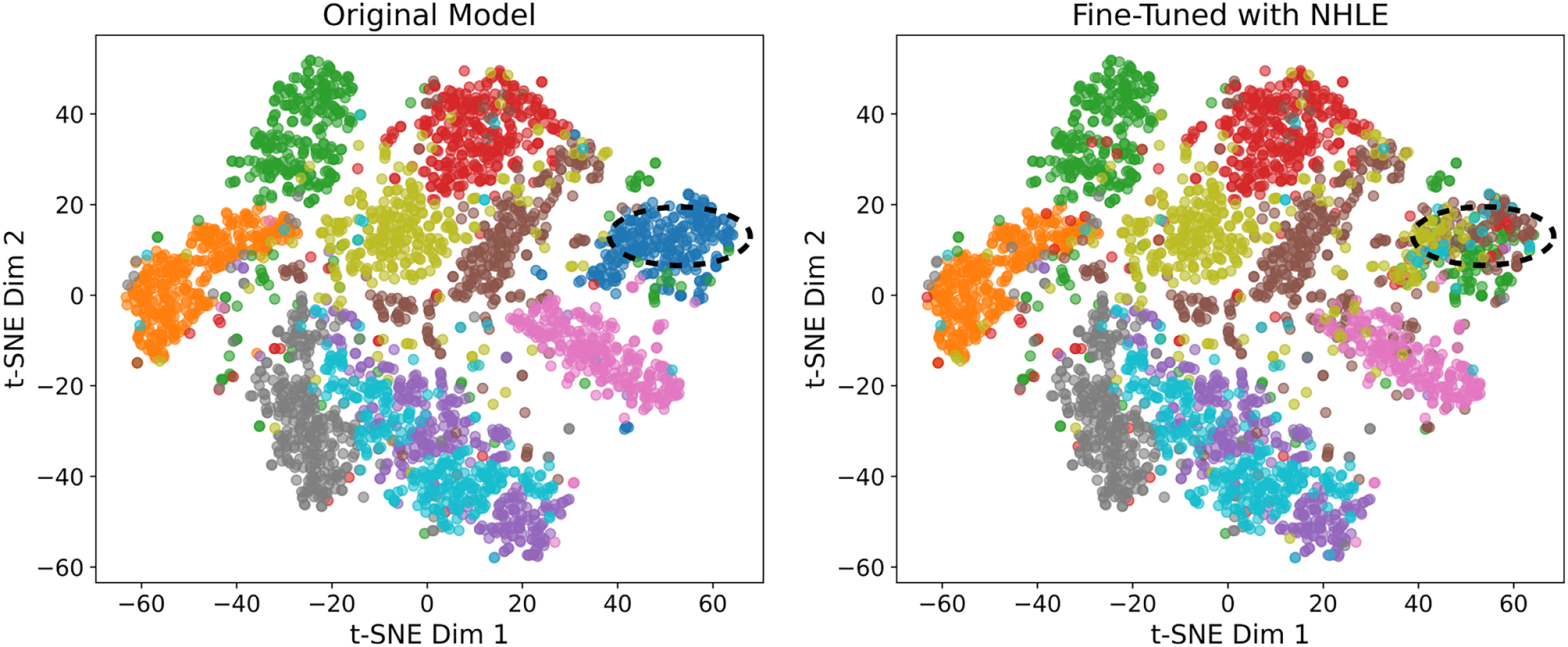
t-SNE visualization of feature embeddings for test samples from the forgotten class, with data points colored by the model’s prediction. Left: The original model correctly classifies most samples as a single class (one color). Right: The fine-tuned model misclassifies the samples into many different classes (a mix of colors), demonstrating effective unlearning.

This experiment employs t-SNE to visualize the predictions of fine-tuned model with NHLE on the MNIST for the forgotten classes. In Fig. 6, the left panel shows the predictions of the original model, while the right panel shows those of the fine-tuned model. Differences between the two sets of predictions are highlighted with a black dashed circle, illustrating how the forgotten classes transition from being easily identifiable to becoming indistinguishable from other classes.

## Discussion

We propose NHLE-CWM, a novel framework for selective machine unlearning that combines Negative-Hot Label Encoding (NHLE) and Class Weight Masking (CWM). NHLE suppresses the discriminability of forgotten classes in the feature space by assigning negative weights to forgotten classes while distributing positive weights across retained classes. CWM complements this by masking class-specific weights at the decision level, preventing reliance on forgotten classes during inference.

Extensive experiments on MNIST, FashionMNIST, SVHN, CIFAR10, and CIFAR100 demonstrate that NHLE-CWM effectively reduces the accuracy of forgotten classes to near zero while preserving or even improving the performance of retained classes. Single-class and multi-class forgetting tasks show that the method achieves thorough unlearning with minimal fine-tuning samples. Feature analyses and t-SNE visualizations confirm that forgotten classes become indistinguishable in the feature space, while the separability of retained classes is preserved.

NHLE-CWM is computationally efficient, does not require access to the full original training set, and provides a scalable, practical solution for controlled and reliable machine unlearning, highlighting the potential of combining label-space manipulation with class-specific weight masking in neural networks.

## Conclusion

We present a machine unlearning framework that selectively eliminates the influence of specific classes from trained neural networks. By integrating strategies that reduce the feature separability of forgotten classes and constrain their impact at the decision layer, the method achieves effective unlearning with only a small number of forgotten samples, while preserving the performance on retained data. Overall, NHLE-CWM offers a practical, reliable, and effective solution for controlled machine unlearning.

## Data Availability

The datasets used in this study are all publicly available: CIFAR-10 and CIFAR-100 [https://www.cs.toronto.edu/kriz/cifar.html], SVHN [http://ufldl.stanford.edu/housenumbers/], Fashion-MNIST [https://github.com/zalandoresearch/fashion-mnist], and MNIST [http://yann.lecun.com/exdb/mnist/]
